# Arthroscopic approach for treating a pigmented villonodular sinovitis of TMJ. A case report

**DOI:** 10.4317/jced.53114

**Published:** 2017-02-01

**Authors:** Maria Roman-Ramos, Paolo Cariati, Almudena Cabello-Serrano, Miguel Garcia-Martin, Blas Garcia-Medina

**Affiliations:** 1 Oral and Maxillofacial surgery resident. Hospital Universitario Virgen de las nieves, Granada, Spain; 2 Maxillofacial Surgeon. Hospital Universitario Virgen de las nieves, Granada, Spain; 3 Dental student. University of Granada

## Abstract

The present report describes the case of a 29-year-old man referred to our service for TMJ pain and progressive reduction of the mouth opening. Differential diagnostics included rheumatologic diseases, monoarthritis and intraarticular lumps. In this line, a face CT scan and a MRI of TMJ were carried out in order to ensure a proper diagnosis. These tests showed a solid lesion into the joint cavity. In view of that, we decided to perform a diagnostic and therapeutic arthroscopy of TMJ. Histopathological studies confirmed the diagnosis of pigmented villonodular synovitis. The main aim of this report is to describe this rare syndrome with the goal of proposing suitable treatments. Moreover, we highlight the benefits of using arthroscopic procedures in the cases which the tumor is still confined to the joint. As far as we are aware, scientific literature documents only a single case of pigmented villonodular synovitis of TMJ treated with arthroscopic approach.

** Key words:**Arthroscopic approach, pigmented villonodular synovitis, TMJ, mouth opening.

## Introduction

Pigmented villonodular synovitis is a rare benign tumor (1). Although the etiology of this disorder is unknown it usually affect the knee (1). However, it may involve the TMJ too. Pigmented villonodular synovitis could be classified into two major groups: a) nodular form; b) diffuse form (representing 80 percent of the cases reported). In this light, several authors reported that the diffuse subtype is considerably more aggressive than the nodular subtype. In fact, in cases of TMJ location it might provoke an injury of the middle cranial fosa (2).

With regard to the diagnosis, is important to highlight that the realization of a histopathologic study is mandatory. Indeed, it represents the best way to ensure an accurate diagnosis.

Surgery is the treatment of choice. Notwithstanding, postoperative radiotherapy may be useful for local control of diffuse form (3).

## Case Report

We describe the case of a 29-year-old man who was referred to our service for TMJ pain and progressive reduction of the mouth opening.

Consequently, anamnesis and physical examination with codified clinical history were carried out. Patient reported chronic TMJ pain (right side) which began six months earlier. The grief was defined as continuous and intolerable. Moreover, any medications was effective in reducing patient pain during the last few months.

In the same line, physical exploration revealed a considerable reduction of the mouth opening (<2,5 cm) with significant difficulty for protrusive and laterotrusive movements. Against this background, we considered three main groups of pathology as possible sources of patient’s symptoms.

1- Rheumatologic diseases such as arthritis rheumatoid, systemic lupus erythematosus and spondylarthrosis.

2- Monoarthritis

3- Intraarticular lumps with progressive tumor growth (synovial hemangioma, synovial chondromatosis, synovial sarcoma, pigmented villonodular synovitis).

Obviously, the absence of systemic symptoms and the duration of episode forced us to consider an intraarticular lumps as the first option for diagnosis.

**Figure 1:**
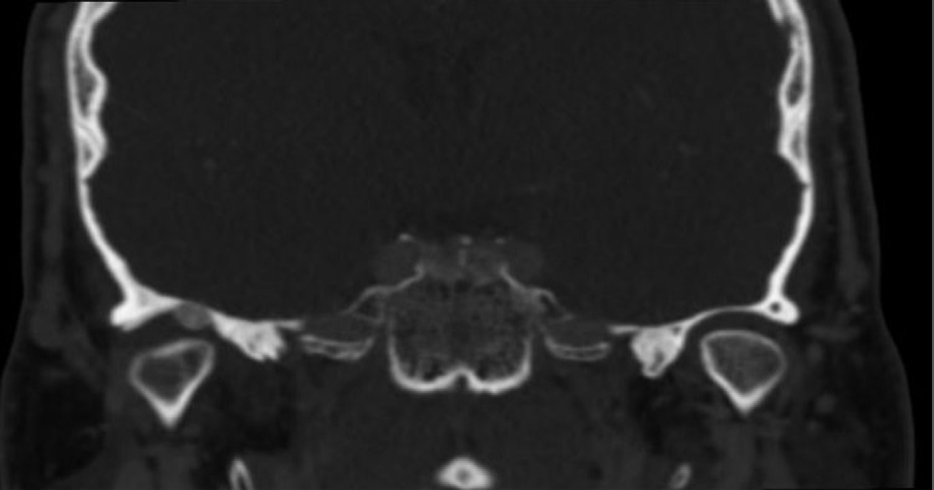
Pigmented villonodular sinovitis of TMJ. CT image (right size).

Thus, we decided to perform a CT scan of the face and a MRI of TMJ. These tests showed the presence of a solid ovoid lesion into the joint cavity (Fig. 1). Importantly, the middle cranial fosa was not affected by the tumor.

**Figure 2:**
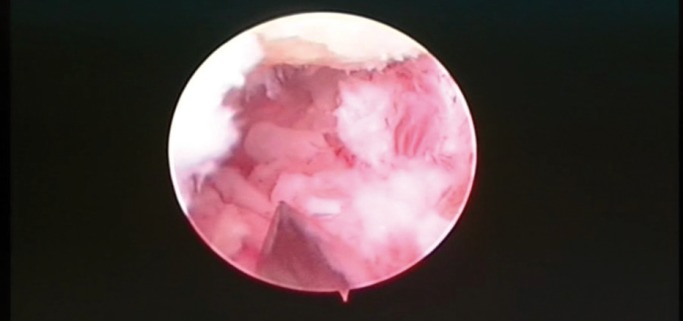
Arthroscopic view of pigmented villonodular synovitis.

**Figure 3:**
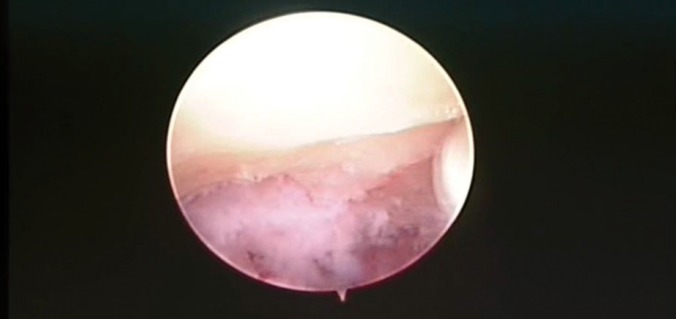
Joint cavity after surgical removal of the tumor.

Finally, we performed a TMJ arthroscopy. The main purpose of surgery was to establish a diagnosis, determine the extent of the disease, and to remove the tumor. The surgical technique was performed using an arthroscopy (2,2 mm) produced by Dyonics. The examination of the joint space was performed on posterioranterior direction with the posterolateral cannula. Arthroscopic view revealed the presence of a multilobal mass into the superior joint space. Specifically, lesion directly affected the joint eminence (Fig. 2). Then, using a triangulation technique, another cannula was introduced in the posterior joint space. The surgical debridement of the tumor was realized through an arthroscopic mill (Fig. 3). Finally, a subsynovial infiltration (with dexamethasone and bupivacaine) in the posterior joint space was performed. A postoperative face CT scan confirmed that the tumor was completely removed and the patient was discharged one day after surgery.

The final diagnosis of villonodular sinovitis was validated by histopathological studies showing an intense proliferation of synovial tissue, hypertrophic synovium cells containing generous amounts of hemosiderin and numerous blood vessels.

A follow-up MRI confirmed the absence of tumour recurrence 16 months after surgery.

## Discussion

Pigmented villonodular sinovitis of TMJ is a rare disorder of unknown aetiology. Only 72 cases have been described in the literature. Early diagnosis and treatment is essential in order to assure an appropriate and effective management of these cases (4,5). Is important to underline that the symptoms of this disease might often be confused with other TMJ disorders such as condromathosis, disc displacement and anchored disk syndrome (6-9). Surgery represent the first option for treating pigmented villonodular sinnovitis (10). Specifically, the total excision of the sinovium is required due to a high risk of recurrence (11,12). In this line, several papers affirms that open surgery is a highly recommended option in order avoid the tumor relapse (8). Moreover, postoperative radiotherapy might represent a strength treatment in patients presenting diffuse subtype or in the cases with incomplete tumor excision. However, there is not enough evidence to assess the benefits and harms of this therapy (3). In our knowledge, there is only a single case report that describe the use of an arthroscopic approach for removing this type of tumor from TMJ space (8). Considering all this, the main aim of the present report is to show the benefits of using an arthroscopic approach to treat pigmented villonodular synovitis. Importantly, we recommended the use of this approach only in the nodular form and in the cases in which the tumor is still confined to the joint. In our point of view, the arthroscopic approach offers numerous advantages. First, it represent a great diagnostic procedure. In fact, we might obtain samples of pathological tissue with minimally surgical invasiveness. Second, we firmly believe that arthroscopic techniques allow to reach an effective eradication of the tumor. Third, this technique guarantees minimal postoperative morbidity.

## References

[1] Damodar D, Chan N, Kokot N (2015). Pigmented villonodular synovitis of the temporomandibular joint: Case report and review of literature. Head Neck.

[2] Chen Y, Cai XY, Yang C, Chen MJ, Qiu T, Zhu Z (2015). J Pigmented villonodular synovitis of the temporomandibular joint with intracranial extension. J Craniofac Surg.

[3] Joshi K, Huang B, Scanga L, Buchman C, Chera BS (2015). Postoperative radiotherapy for diffuse pigmented villonodular synovitis of the temporomandibular joint. Am J Otolaryngol.

[4] Kim IK, Cho HI, Cho HW, Seo JH, Lee DH, Peng W (2014). Pigmented villonodular synovitis of the temporomandibular joint -computed tomography and magnetic resonance findings: a case report. J Korean Assoc Oral Maxillofac Surg.

[5] Safaee M, Oh T, Sun MZ, Parsa AT, McDermott MW, El-Sayed IH (2014). Pigmented villonodular synovitis of the temporomandibular joint with intracranial extension: A case series and systematic review. Head Neck.

[6] Le WJ, Li MH, Yu Q, Shi HM (2014). Pigmented villonodular synovitis of the temporomandibular joint: CT imaging findings. Clin Imaging.

[7] Giannakopoulos H, Chou JC, Quinn PD (2013). Pigmented villonodular synovitis of the temporomandibular joint. Ear Nose Throat J.

[8] Cai XY, Yang C, Chen MJ, Jiang B, Yun B, Fang B (2011). Arthroscopic management of intra-articular pigmented villonodular synovitis of temporomandibular joint. Int J Oral Maxillofac Surg.

[9] Reñaga Rubin I, Salavert Girona A, Vasquez Rodriguez A, Anmella Valmanya J (1997). Pigmented villonodular syno- vitis of the temporomandibular joint. Oral Surg Oral Med Oral Pathol Oral Radiol Endod.

[10] Aoyama S, Iwaki H, Amagasa T, Kino K, Okada N, Kishimoto S (2004). Pigmented villonodular synovitis of the temporo- mandibular joint: differential diagnosis and case report. Br J Oral Maxillofac Surg.

[11] Cascone P, Filiaci F, Paparo F, Mus- Tazza MC (2008). Pigmented villonodular synovitis of the temporomandibular joint. J Orofac Pain.

[12] Aimoni C, Ciorba A, Cappiello L, Giuriato R, Denes SA, Galiè M (2012). Pigmented villonodular synovitis of the temporomandibular joint. J Craniofac Surg.

